# Supplementary material for: Controllable gliders in a nanomagnetic metamaterial
Source: Nat Commun. 2025 Aug 13;16:7500. doi: 10.1038/s41467-025-62515-1 (PMC12350634; doi:10.1038/s41467-025-62515-1)
Supplement: Supplementary file 1 — Supplementary Information [file 41467_2025_62515_MOESM1_ESM.pdf]

Supplementary information: Controllable Gliders in a Nanomagnetic Metamaterial

Arthur Penty[1*], Johannes H. Jensen[1], Ida Breivik[2], Anders Strømberg[2], Erik Folven[2], Gunnar Tufte[1]

[1]Department of Computer Science, Norwegian University of Science and Technology, Trondheim, Norway.
[2]Department of Electronic Systems, Norwegian University of Science and Technology.

*Corresponding author(s). E-mail(s): arthur.penty@ntnu.no;

1 Supplementary Discussion

1.1 MuMax3 simulations

To assess the viability of experimental realisations, the snake was first verified in micromagnetic simulations using MuMax3 [1]. Figure 1 shows snapshots from the MuMax3 simulations, where a leftwards snake is successfully realised. As can be seen, a 10×10 pinwheel ASI is first initialised with a snake in the centre, as shown in Figure 1 (0). Then the $bBaA$ clock protocol is applied, for two clock cycles, as shown in Figure 1 (1-8), using $H^+ = 34\,\mathrm{mT}$ and $H^- = 33\,\mathrm{mT}$. The strength of each clock field was increased from zero to the target field strength using a ramp function $\tanh(15ft)$ with $f = 10^8$. The ramp function results in an initially rapid increase of the field strength, followed by a slow approach towards the target field strength for a duration of 10 ns. Ramp duration was found to be sufficient to allow the nanomagnets' internal spin wave dynamics to settle between each field application. The simulated nanomagnets are stadium-shaped with dimensions $220\,\mathrm{nm} \times 80\,\mathrm{nm} \times 10\,\mathrm{nm}$, arranged with a lattice spacing of 280 nm. Simulation parameters include saturation magnetisation $M_\mathrm{S} = 860\,\mathrm{kA\,m^{-1}}$, exchange stiffness $A_\mathrm{ex} = 1.3 \times 10^{-11}\,\mathrm{J\,m^{-1}}$, damping constant $\alpha = 0.01$, a world size of 1024×1024 cells and a cell size of $5\,\mathrm{nm} \times 5\,\mathrm{nm} \times 10\,\mathrm{nm}$.

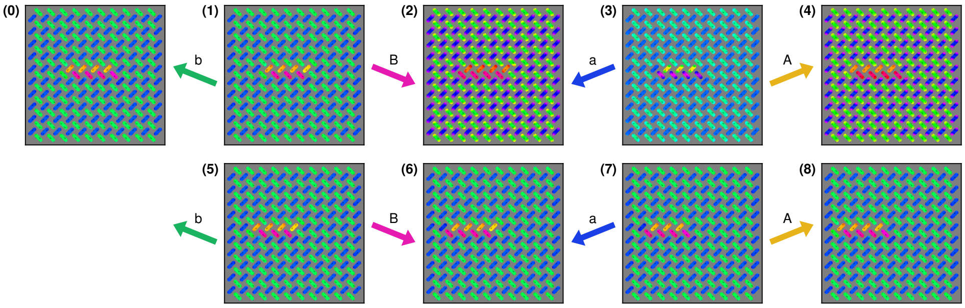

Fig. 1: MuMax3 simulations of a snake in 10×10 pinwheel ASI. (0) shows the initial state of the snake. (1-8) shows the state of the snake during $bBaA$ clocking. The field arrow to the left of each snapshot indicate the clock field which precedes it. A video of the simulation is provided in Supplementary Movie 3.

1.2 Experimental realisation of clocking with equal strength clock fields

To experimentally verify that H^- must be strictly less than H^+, we polarise and write a new initial snake state, as shown in Figure 2 (0). We then find H^+ by applying the B clock field with increasing field strength, until the snake grows, as shown in Figure 2 (1). The B field is used to find H^+ because, in this particular written initial state, the next unlocked nanomagnet at the head of the snake is in sublattice L_b. When we apply the next clock field, a, with $H^- = H^+ = 21\,\text{mT}$, almost the entire snake state disappears, as shown in Figure 2 (2). To verify that this effect is not due to variations in the switching fields of the nanomagnets, we repeat this process for two more snakes in different parts of the array.

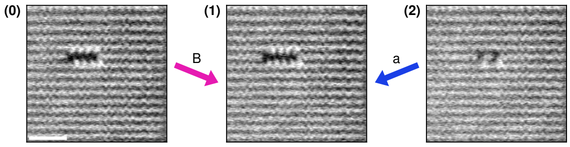

Fig. 2: Experimental clocking of a snake with $H^- = H^+$. MFM-micrographs of part of a 100×100 pinwheel ASI. (0) shows the initial state of the snake. H^+ is found by applying the B field with increasing field strength until the snake grows (1). In (2), the a field is applied with $H^- = H^+ = 21\,\text{mT}$, resulting in most of the nanomagnets in the snake switching, making the snake state disappear.

1.3 Monopoles and magnetic charge visualisation

When looking for moving structures in ASI, one might naturally think of monopole dynamics, as seen in square and kagome ASI [2, 3]. However, for our purposes, we

do not consider monopole dynamics as a type of translation. Despite the free movement of monopoles in the unconfined regime [4, 5], they are topologically limited by Dirac strings. These Dirac strings are connections from a monopole to the edge of the ensemble or some other defect [3, 6], or another oppositely charged monopole which by symmetry of charge will move in the opposite direction [7]. Such monopole dynamics result in an extension or retraction of the structure (e.g., growing and shrinking a Dirac string) rather than translation.

The emergent monopole picture is still a powerful tool, and it might be instructive to view the snake glider in the context of magnetic charge. The vertices in Pinwheel ASI are conventionally not considered to exhibit emergent monopoles. However, we can attribute some 'magnetic charge' to vertices in ferromagnetic domain walls in Pinwheel ASI by calculating whether vertices have a net surplus of magnetic moment directly into or out of the vertex center [8].

The magnetic charge visualisation for two opposite moving snakes is shown in Figure 3, and provides some insight into the nature of the snake glider. Each snake consists of a vortex-antivortex pair, with the vortex at the head and the antivortex at the tail. Along the body of the snake there are pairs of partial magnetic charges, $\pm 2q$. The vortex at the head has a full charge, $\pm 4q$, which we term a 'monopole'. As fields are applied, the monopole heads move with or against the A and B fields according to the sign of their charge, just as we expect magnetic charges would. The monopoles are affected by the larger A and B fields but are unaffected by the smaller a and b fields. The a and b fields shrink the snake by annihilating a pair of partial $\pm 2q$ charges at the tail of each snake, and moving the backmost partial charge into the antivortex. This charge picture explains well the difference between the two types of snakes and which direction each snake will move with an applied field.

However, there are several aspects the magnetic charge picture does not explain well. It is not clear why the partial charges of the tail are more affected by the smaller-magnitude shrinking field, and not by the growing fields. The magnetic charge picture is also completely ignoring the relevance of the precise field angles, which can only be accounted for by including the switching astroid. While there are some intuitive lessons to be learned from this perspective, it is ultimately an abstraction level that falls short of explaining the phenomenon in detail.

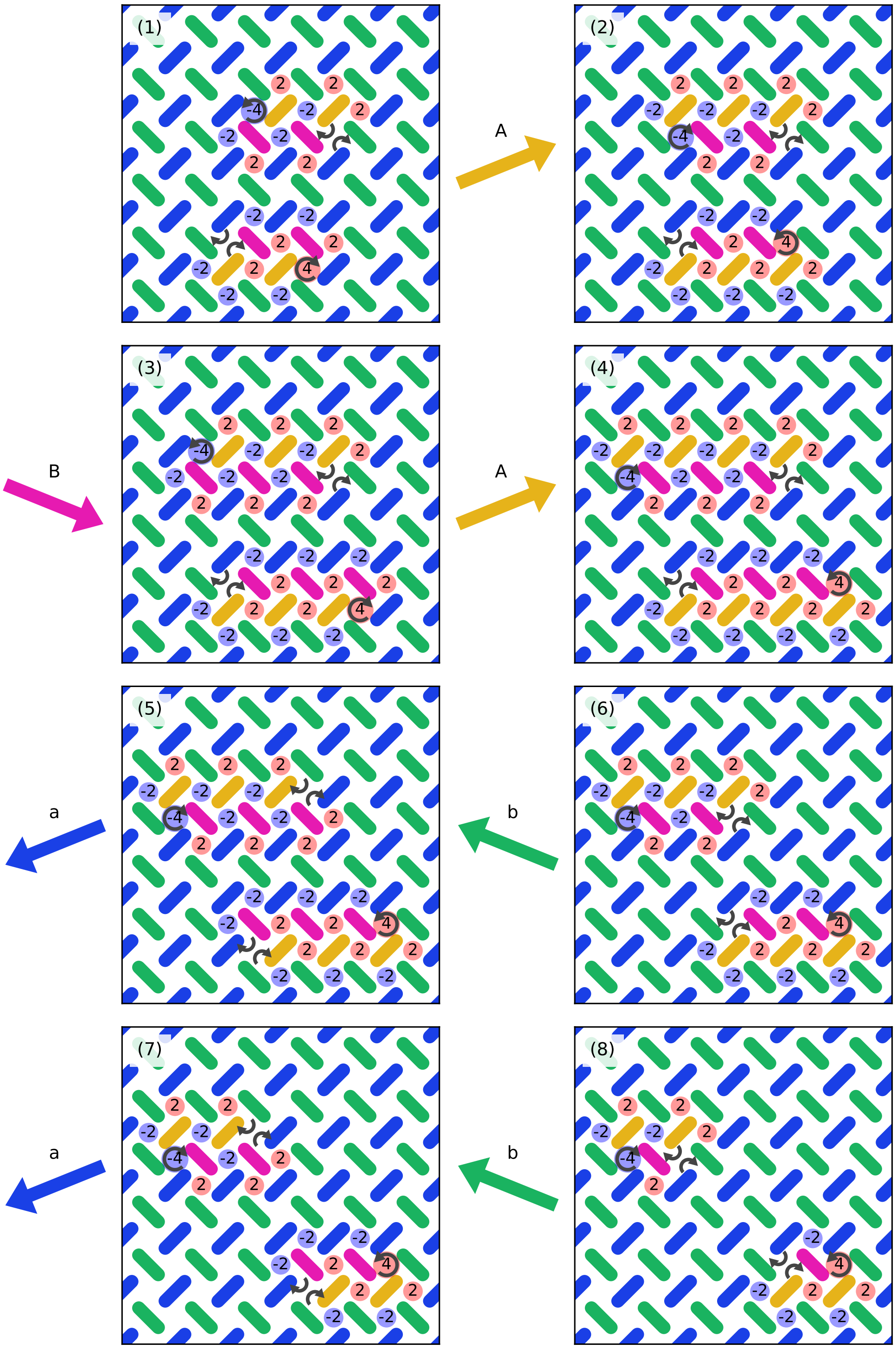

Fig. 3: Leftwards and rightwards moving snake gliders in pinwheel ASI with partial charges indicated at each pinwheel vertex. Each snapshot shows a zoomed-in view of a 12×12 system, at different points during AB (2-4) and ab (5-8) clock protocols. (1) shows the initial state of the snakes. Positive (negative) charges are indicated as red (blue) circles, labelled according to the sum of the number of heads with an inwards component with respect to the vertex center. The grey curved arrows indicate vortices and antivortices. The field arrow to the left of each snapshot indicates the clock field which precedes it.

2 Supplementary Figures

2.1 Leftwards and rightwards moving snakes

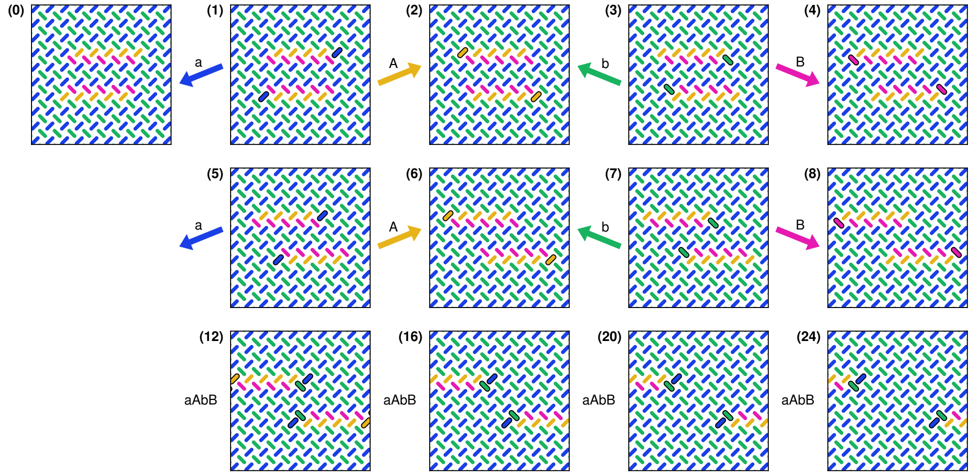

Fig. 4: Leftwards and rightwards moving snake gliders in pinwheel ASI. Each snapshot shows a zoomed-in view of a 50×50 system, at different points during the $aAbB$ clock protocol. (0) shows the initial state of the snakes, two elongated orange/pink domains in the centre of a blue/green background domain. (1-8) shows the state of the snakes during $aAbB$ clocking, where magnets that change state between snapshots are highlighted by a solid black outline. The field arrow to the left of each snapshot indicates the clock field which precedes it. The top snake moves leftwards, while the bottom snake moves rightwards, through alternate shrinking (a and b) and growing (A and B) steps. The bottom row continues the series, showing only snapshots after a complete clock cycle (four clock fields are applied between each snapshot). A video of the simulation is provided in Supplementary Movie 2.

2.2 Neighbour influence analysis for all fields

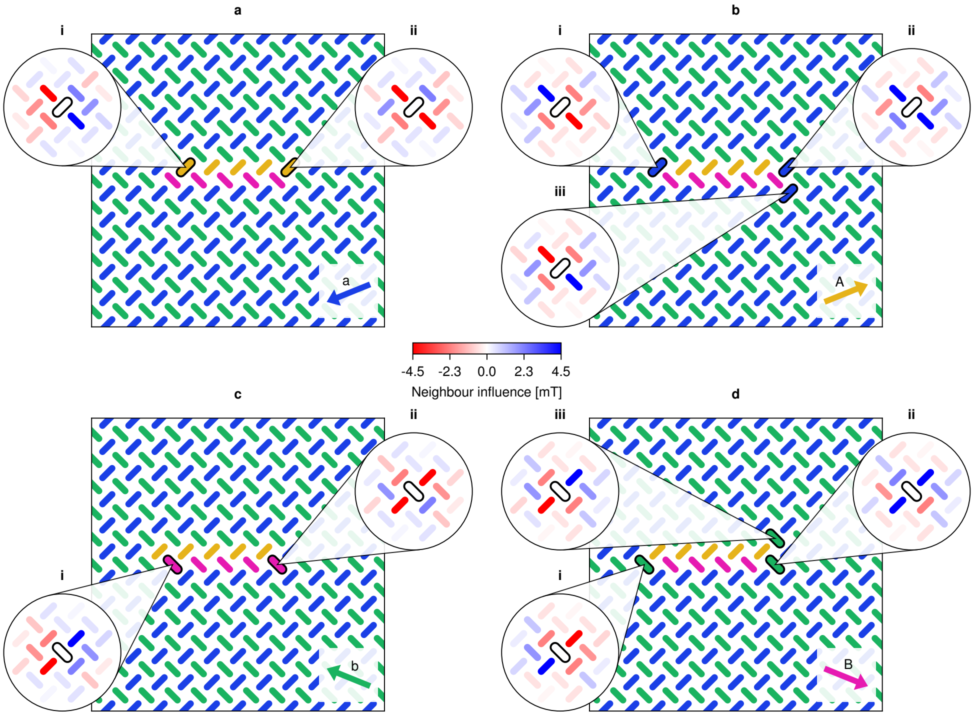

Fig. 5: Neighbour influence during **a** shrinking of L_a, **b** growth of L_a, **c** shrinking of L_b and **d** growth of L_b. The insets depict the switching influence from the neighbours of the highlighted magnet. Stronger red signifies that the magnet is biasing the highlighted magnet towards switching, and stronger blue signifies that the magnet is biasing the highlighted magnet away from switching. Notice how the switching influences for **c** are mirror images of **a**, and similarly for **d** and **b**.

2.3 Astroid clusters

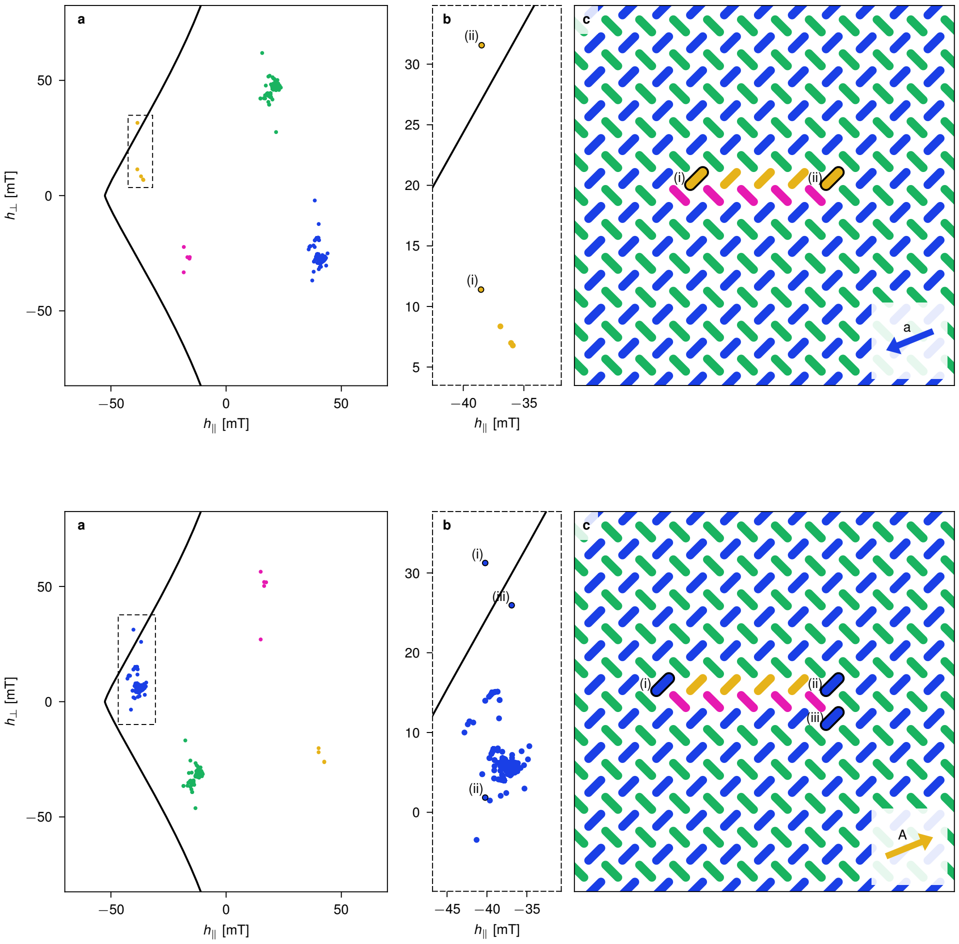

Fig. 6: Astroid clusters showing relative locations of all the magnets within their respective switching astroids, when subject to the clock fields a (top) and A (bottom). The plots **a-b** show the astroid clusters, where each dot represents the total field experienced by a magnet, projected onto its parallel ($h_{\|}$) and perpendicular ($h_{\perp}$) axis. Note that the positive direction of the parallel component is with respect to the magnetisation direction of each nanomagnet. The highlighted magnets in **c** correspond to the marked dots in **b**, supplementing the influence plots from Figure 5a-b.

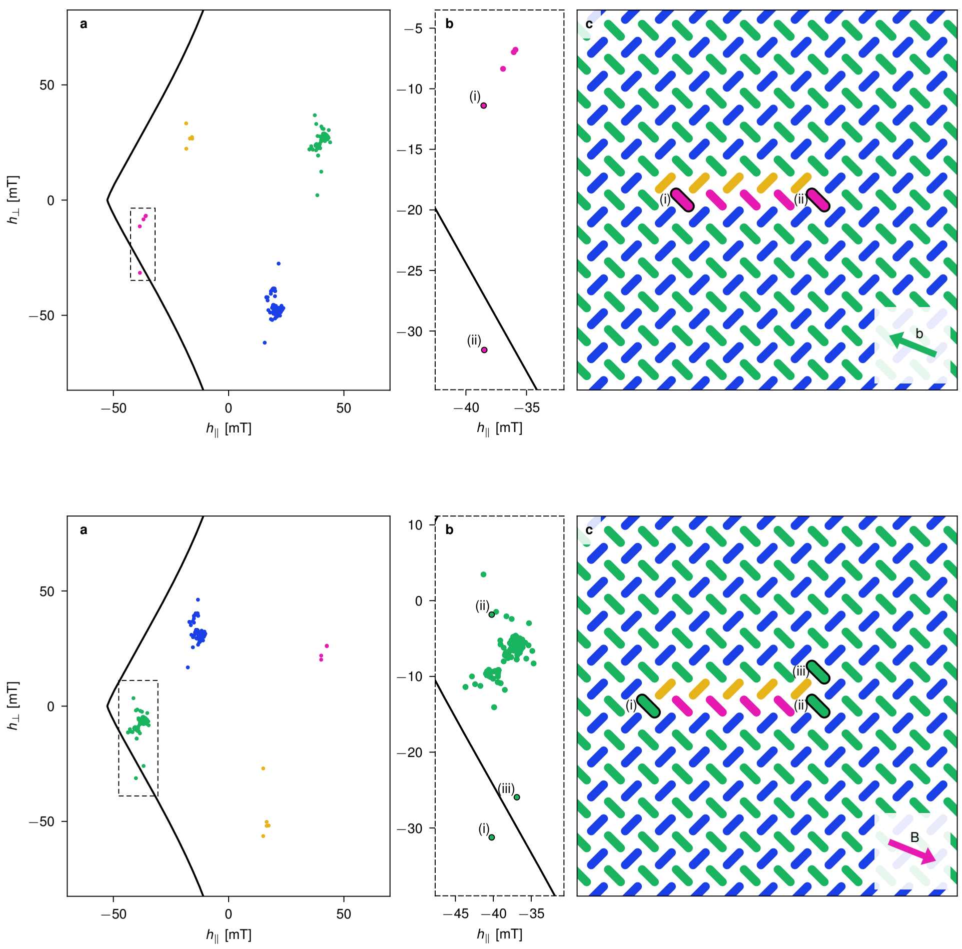

Fig. 6 (cont.): Astroid clusters showing relative locations of all the magnets within their respective switching astroids, when subject to the clock fields b (top) and B (bottom). The plots **a-b** show the astroid clusters, where each dot represents the total field experienced by a magnet, projected onto its parallel ($h_{||}$) and perpendicular ($h_{\perp}$) axis. Note that the positive direction of the parallel component is with respect to the magnetisation direction of each nanomagnet. The highlighted magnets in **c** correspond to the marked dots in **b**, supplementing the influence plots from Figure 5c-d.

2.4 Other Gliders

Figures 7 to 9 show some other gliders that were discovered by the evolutionary run. In all of these cases we see more complex reversal-dynamics than in the snake, the clock fields can flip multiple magnets in the same step, and even magnets on different

sublattices. Due to this increased complexity and the additional difficulty of realising the structures experimentally, we instead focussed on the simpler and more promising snake glider.

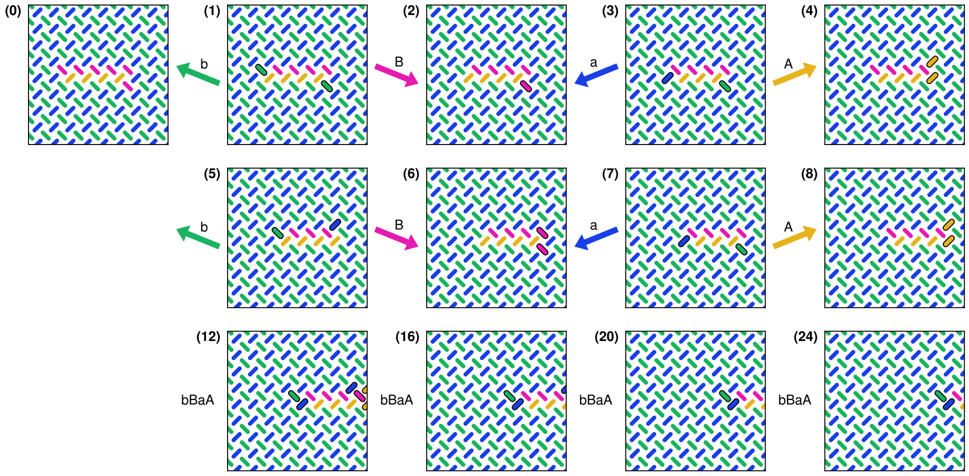

Fig. 7: “Horned Snake” glider discovered through evolution.

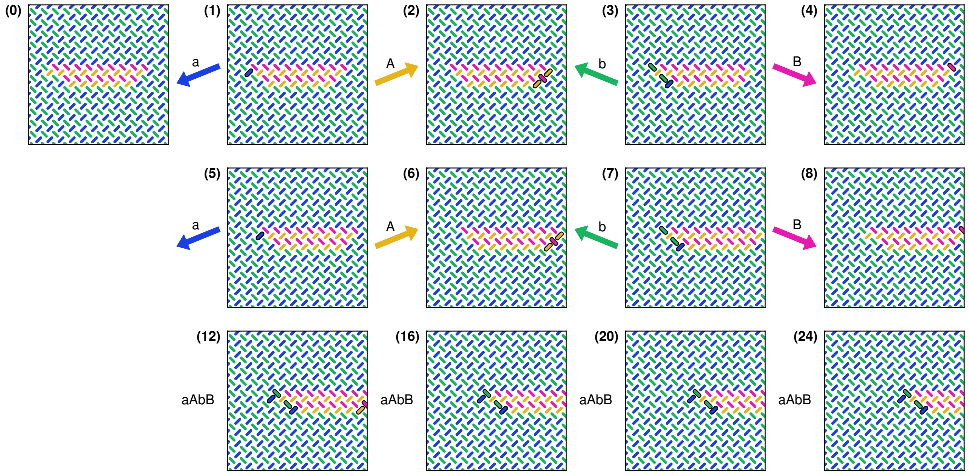

Fig. 8: “Slug” glider discovered through evolution.

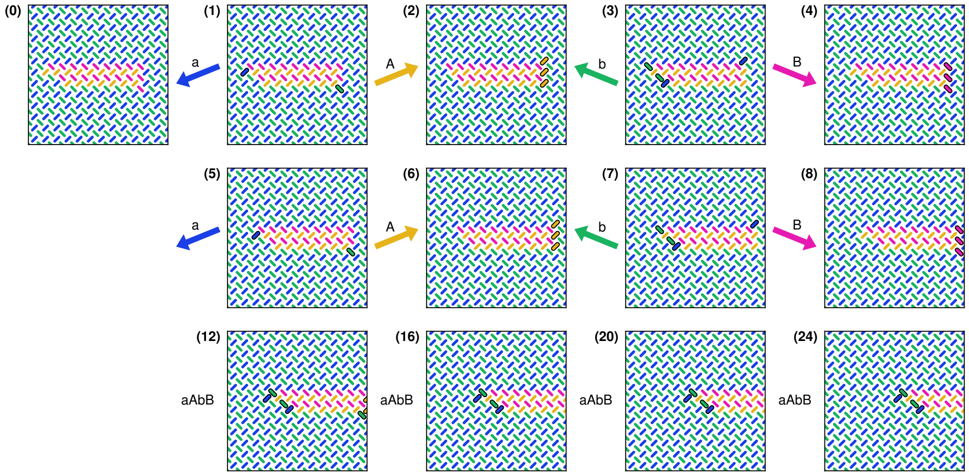

Fig. 9: "Horned Slug" glider discovered through evolution.

References

[1] Vansteenkiste, A., Leliaert, J., Dvornik, M., Helsen, M., Garcia-Sanchez, F., Van Waeyenberge, B.: The design and verification of MuMax3. AIP Advances **4**(10), 107133 (2014) https://doi.org/10.1063/1.4899186

[2] Mengotti, E., Heyderman, L.J., Rodríguez, A.F., Nolting, F., Hügli, R.V., Braun, H.-B.: Real-space observation of emergent magnetic monopoles and associated Dirac strings in artificial kagome spin ice. Nature Physics **7**(1), 68–74 (2011) https://doi.org/10.1038/nphys1794

[3] Arava, H., Vedmedenko, E.Y., Cui, J., Vijayakumar, J., Kleibert, A., Heyderman, L.J.: Control of emergent magnetic monopole currents in artificial spin ice. Physical Review B **102**(14), 144413 (2020) https://doi.org/10.1103/PhysRevB.102.144413

[4] Nascimento, F.S., Mól, L.A.S., Moura-Melo, W.A., Pereira, A.R.: From confinement to deconfinement of magnetic monopoles in artificial rectangular spin ices. New Journal of Physics **14**(11), 115019 (2012) https://doi.org/10.1088/1367-2630/14/11/115019

[5] Farhan, A., Saccone, M., Petersen, C.F., Dhuey, S., Chopdekar, R.V., Huang, Y.-L., Kent, N., Chen, Z., Alava, M.J., Lippert, T., Scholl, A., van Dijken, S.: Emergent magnetic monopole dynamics in macroscopically degenerate artificial spin ice. Science Advances **5**(2), 6380 (2019) https://doi.org/10.1126/sciadv.aav6380

[6] Puttock, R., Andersen, I.M., Gatel, C., Park, B., Rosamond, M.C., Snoeck, E., Kazakova, O.: Defect-induced monopole injection and manipulation in artificial spin ice. Nature Communications **13**(1), 3641 (2022) https://doi.org/10.1038/s41467-022-31309-0

[7] Loreto, R.P., Morais, L.A., Silva, R.C., Nascimento, F.S., Araujo, C.I.L., Mól, L.A.S., Moura-Melo, W.A., Pereira, A.R.: Realization of Magnetic Monopoles Current in an Artificial Spin Ice Device: A Step towards Magnetronics. arXiv (2014). https://doi.org/10.48550/arXiv.1404.4082

[8] Li, Y., Paterson, G.W., Macauley, G.M., Nascimento, F.S., Ferguson, C., Morley, S.A., Rosamond, M.C., Linfield, E.H., MacLaren, D.A., Macêdo, R., Marrows, C.H., McVitie, S., Stamps, R.L.: Superferromagnetism and domain-wall topologies in artificial “Pinwheel” spin ice. ACS Nano **13**(2), 2213–2222 (2019) https://doi.org/10.1021/acsnano.8b08884
