# Supplementary material for: Controllable gliders in a nanomagnetic metamaterial
Source: Nat Commun. 2025 Aug 13;16:7500. doi: 10.1038/s41467-025-62515-1 (PMC12350634; doi:10.1038/s41467-025-62515-1)
Supplement: Supplementary file 2 — Description of Additional Supplementary Files [file 41467_2025_62515_MOESM2_ESM.pdf]

Description of Additional Supplementary Files

Supplementary Movie 1: flatspin simulation showing 40 steps of snake movement (10 full clock cycles). A labelled arrow on the left shows the most recently applied clocking field.

Supplementary Movie 2: flatspin simulation showing two snakes, of opposing direction, undergoing 40 steps of snake movement (10 full clock cycles).

Supplementary Movie 3: MuMax3 simulation demonstrating eight steps of snake movement.

Supplementary Movie 4: MFM-micrographs of the experimental demonstration of the snake. The initial snake state (green) is written using an MFM-tip in the presence of a 10 mT bias field. To optimize the clock field strengths we then apply a series of global fields along the clock directions (blue). The snake is then moved two whole clock cycles (white). In the third clock cycle (pink), the tail of the snake explodes. The scale bar is 1 μm.

Supplementary Movie 5: MFM-micrographs of groups of five to six snakes, clocked at H+ = 21.75 mT and H− = 19.25 mT. The snakes are imaged after each full clock cycle. White triangles indicate the rows on which the first group of five snakes is initialized, and the following five groups are initialized on rows above these, as indicated in the movie. In the two series marked in blue, we have initialized the snakes on the same rows of the pinwheel ASI, and subsequently clocked them using the same fields.

Supplementary Movie 6: MFM-micrographs of groups of five to six snakes, clocked at H+ = 21.25 mT and H− = 19.25 mT. The snakes are imaged after each full clock cycle. White triangles indicate the rows on which the first group of five snakes is initialized, and the following five groups are initialized on rows above these, as indicated in the movie.

Supplementary Movie 7: MFM-micrographs of groups of six snakes. The snakes were initialized on the rows marked with white triangles and subsequently clocked with H− = 19.25 mT and H+ = 21.25 mT, 21.5 mT, 21.75 mT, 22 mT. The snakes are imaged after every full field cycle.

Supplementary Movie 8: flatspin simulation of a simple single bit information encoding. A single bit of information is encoded by the presence (or absence) of a length two snake at the far left of the ASI. The ASI is then clocked two full cycles to allow the next bit to be input.

Supplementary Movie 9: flatspin simulation of a simple byte (8-bit) information encoding. Eight bits of information are encoded in parallel by the presence (or absence) of a length two snakes at the far left of the ASI, separated vertically by a gap of six magnets. The ASI is then clocked two full cycles to allow the next byte to be input. An the corresponding base 10 integer is shown.

Supplementary Movie 10: flatspin simulations of snakes colliding. Simulations are shown for a range of different set of H+, H− field strengths. Each simulation shows two separate collisions. In the upper collision, the rightwards moving snake is one row higher than the leftwards moving snake. In the lower collision, the rightwards moving snake is one row lower than the leftwards moving snake.

Supplementary Movie 11: flatspin simulations of almost colliding snakes (one row of separation). Simulations are shown for a range of different set of H+, H− field strengths. Each simulation shows two separate interactions. In the upper interaction, the rightwards moving snake is one row higher

than the leftwards moving snake. In the lower interaction, the rightwards moving snake is one row lower than the leftwards moving snake.
